# Supplementary material for: Bariatric surgery induces a new gastric mucosa phenotype with increased functional glucagon-like peptide-1 expressing cells
Source: Nat Commun. 2021 Jan 4;12:110. doi: 10.1038/s41467-020-20301-1 (PMC7782689; doi:10.1038/s41467-020-20301-1)
Supplement: Supplementary file 1 — Supplementary Information [file 41467_2020_20301_MOESM1_ESM.pdf]

Supplementary information

Bariatric Surgery induces a new gastric mucosa phenotype with increased functional Glucagon-Like Peptide-1 expressing cells

Lara Ribeiro-Parenti[1,2#], Anne-Charlotte Jarry[1#], Jean-Baptiste Cavin[1], Alexandra Willemetz[1], Johanne Le Beyec[1,3], Aurélie Sannier[4], Samira Benadda[1,5], Anne-Laure Pelletier[1], Muriel Hourseau[4], Thibaut Léger[6,7], Bastien Morlet[6], Anne Couvelard[1,4], Younes Anini[8], Simon Msika[1,2], Jean-Pierre Marmuse[1,2], Séverine Ledoux[1,9], Maude Le Gall[1@], André Bado[1@]

[#] *equal contribution*

[1] Université de Paris, Inserm U1149, Centre de recherche sur l'inflammation, Paris, France

[2] Service de chirurgie digestive œsogastrique et bariatrique, Hôpital Bichat - Claude-Bernard, Assistance Publique-Hôpitaux de Paris, Paris, France

[3] Sorbonne Université, AP-HP, Hôpital Pitié-Salpêtrière-Charles Foix, Biochimie Endocrinienne et Oncologique, Paris, France

[4] Department of Pathology Bichat Hospital, AP-HP, F-75018 Paris, France

[5] Cell and Tissue Imaging Platform, Inserm, U1149, CNRS, ERL8252, F-75018 Paris, France.

[6] Université de Paris, Mass Spectrometry Laboratory, Institut Jacques Monod, UMR 7592, CNRS, F-75205 Paris, France

[7] Univ Rennes, Inserm, EHESP, Irset (Institut de recherche en santé, environnement et travail) - UMR_S 1085, F-35000 Rennes, France

[8] Department of Obstetrics and Gynecology, Dalhousie University, IWK Health Centre

[9] Service des Explorations Fonctionnelles Hôpital Louis Mourier, AP-HP, Centre Intégré Nord Francilien de prise en charge de l'Obésité (CINFO), F-92701, Colombes, France.

Running title:
Gastric cells expressing GLP-1

[@] **Correspondence:**
André BADO or Maude LE GALL
INSERM, UMR 1149 – CRI
UFR de Médecine Paris Diderot,
16 rue Henri Huchard,
75890 Paris Cedex 18, France
E-mail: andre.bado@inserm.fr or maude.le-gall@inserm.fr

Supplementary Figure 1

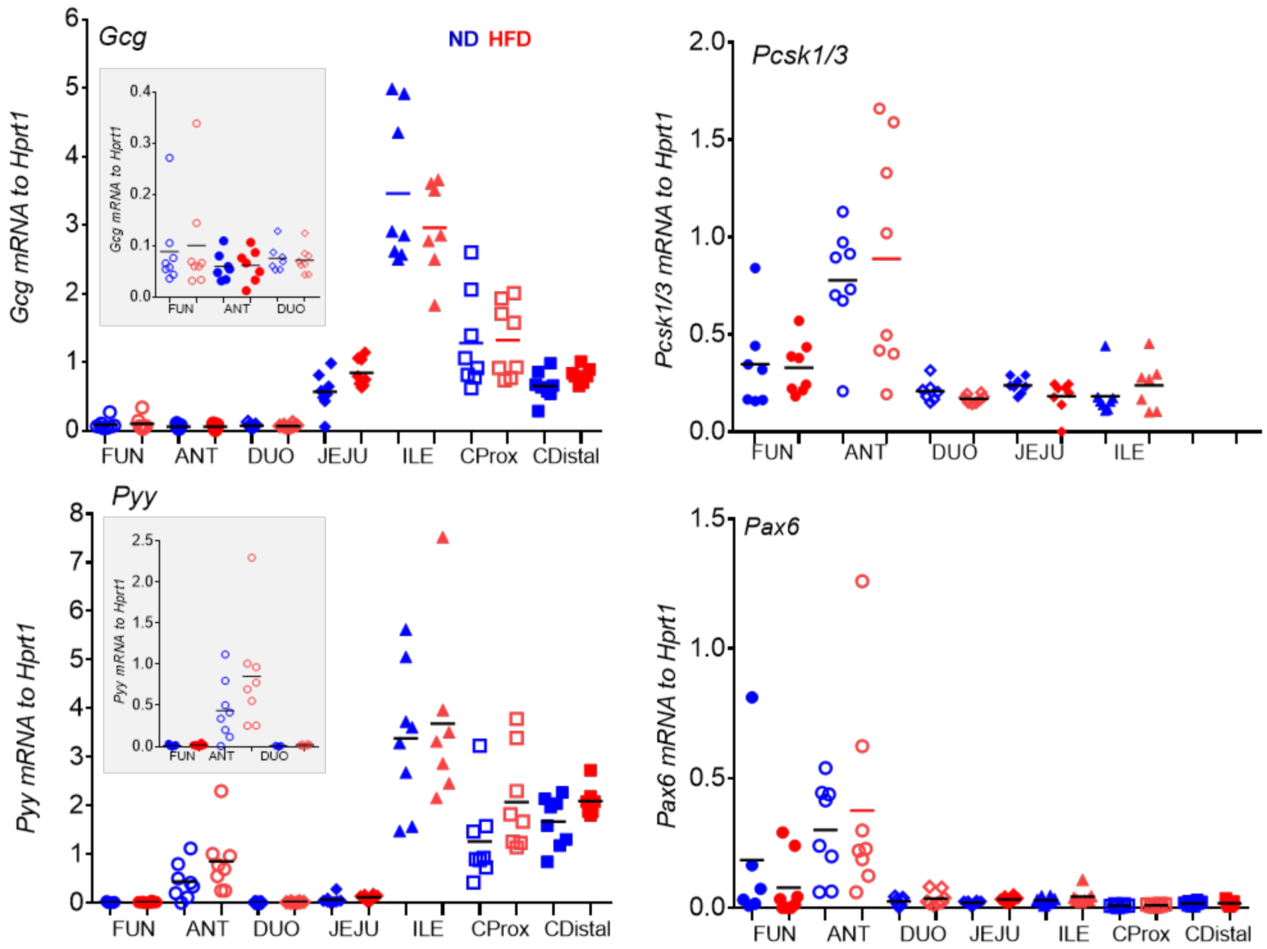

Expression of *preproglucagon* (*Gcg*), *Peptide yy* (*Pyy*), *Pcsk1/3* and *Pax-6* along the gastrointestinal tract in rats fed normal diet (ND blue) or High Fat Diet (HFD red) during 4 months. Expression was normalized to *Hprt1* and each point corresponds to one rat sample. Insert a zoom on mRNA levels in the gastric mucosa. FUN for fundus, ANT for antrum, DUO for duodenum, JEJU for jejunum, ILE for ileum, CProx for proximal colon and CDistal for distal colon

Supplementary Figure 2

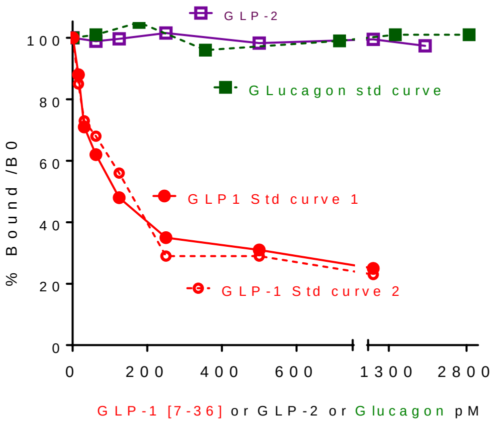

Specificity of GLP-1 radioimmune assay. Representative standard curves of Glucagon-Like Peptide-1 (GLP-1) [7-36] amide (Red), or GLP-2 (purple) or glucagon [7-36] (green) calculated as percent Bound [B]/ Total Binding [B_0]. The antibody used is directed to the C-terminal portion of GLP-1 and recognizes both all amidated and non-amidated forms of GLP- 1. There is no cross-reactivity of glucagon and GLP-2 in this RIA.

Supplementary Figure 3

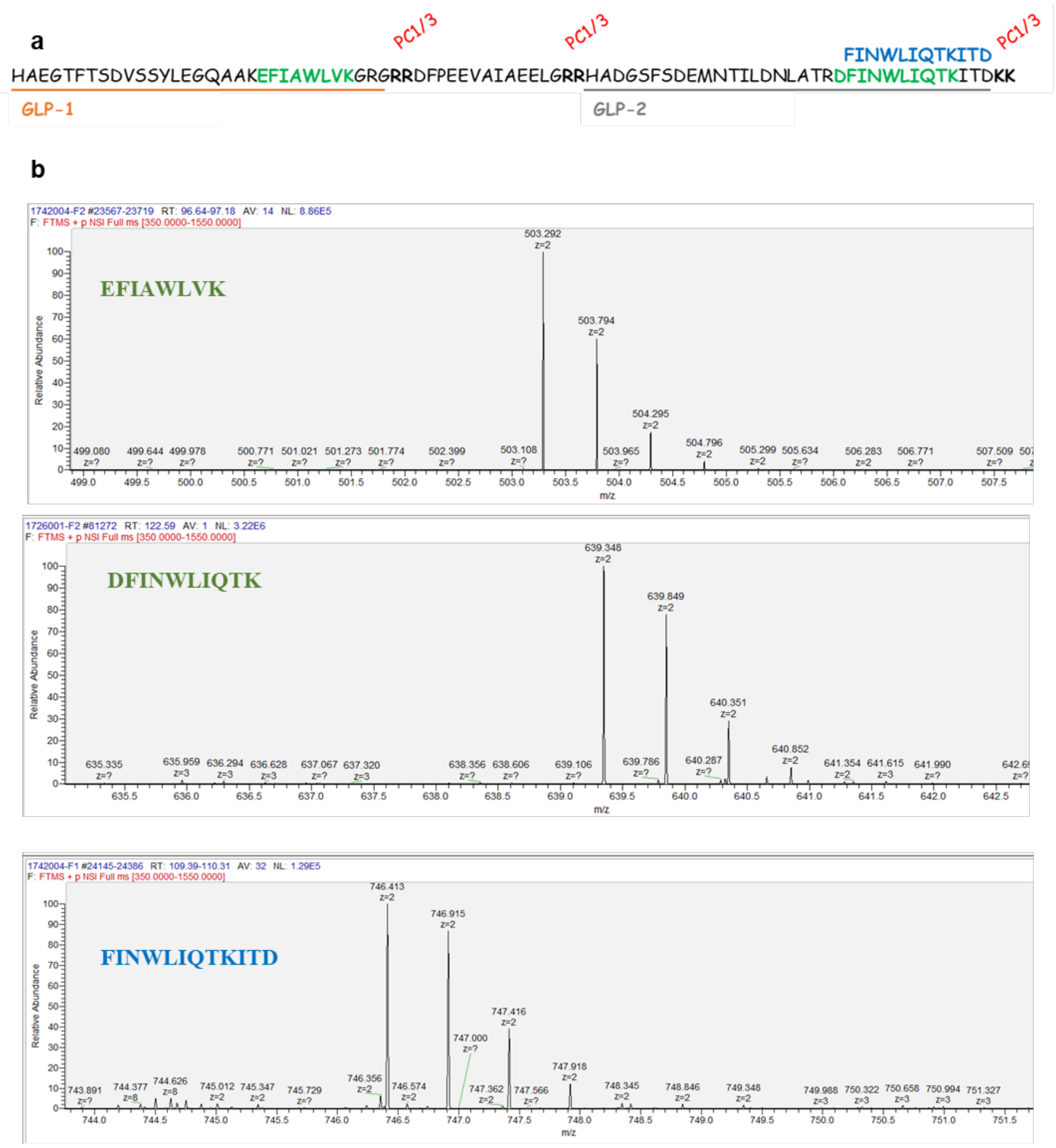

Identification of GLP-1 by LC-MS/MS. [a] Principal peptides derived from GCG, corresponding to GLP-1 or GLP-2, identified in acid-ethanol gastric mucosa extracts from the rat stomach obtained by trypsin and Glu-C digestion, are represented in green and blue, respectively. Physiological cleavage sites by PC1/3 are shown.

[b] Corresponding MS spectrum showing the isotopic mass of the precursor selected for the MS/MS are represented.

Supplementary Figure 4

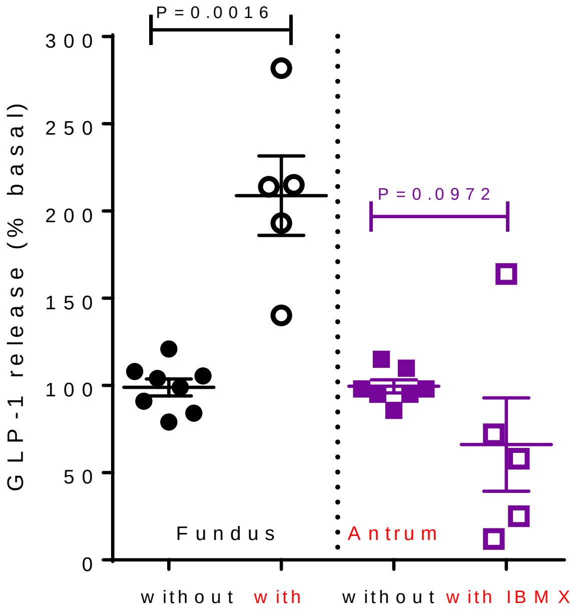

IBMX-stimulated GLP-1 release *ex vivo* from fundus and antrum fragments from HFD obese rats. Overnight fasted rat stomachs were collected and incubated for 1 hour with PBS (without IBMX) and with 100µM IBMX. GLP-1 was assayed in the supernatant and the results are expressed as percent of CTRL (without IBMX). The data are presented as scatter data plots (each point represents one rat) with mean ± SEM and analyzed using Mann-Whitney test. For the Fundus n=8 without and n= 5 with IBMX. For the antrum n=7 without and n= 5 with IBMX.

Supplementary Figure 5

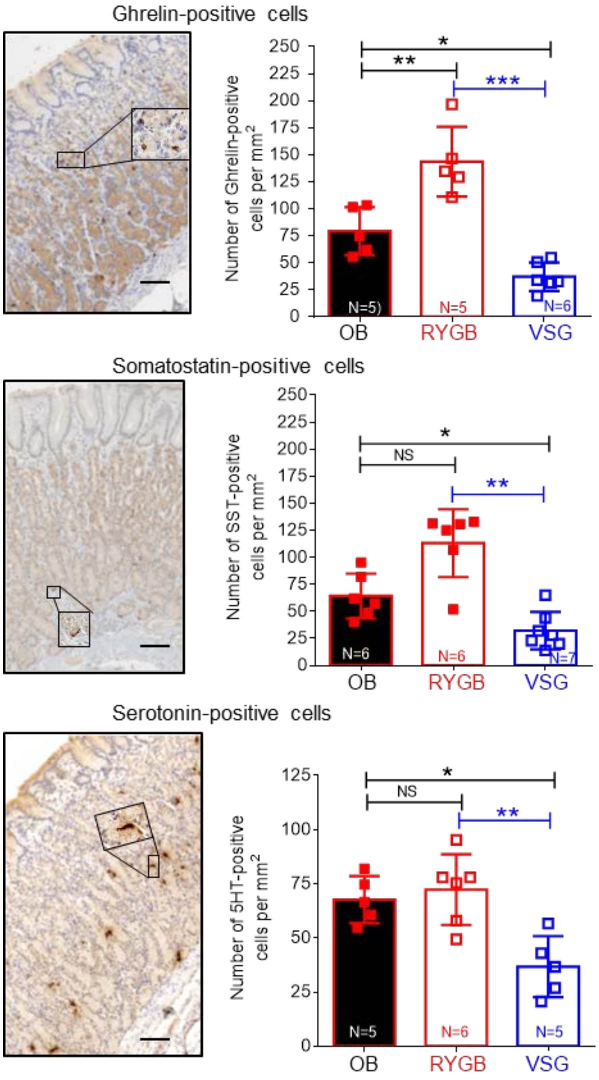

Immunoreactivity of ghrelin-, somatostatin- and serotonin-positive cells in human fundic mucosa from obese subjects operated on RYGB or VSG. Representative Immunostaining (Bar scale 100µm) and quantification of ghrelin-, somatostatin- and serotonin-immunoreactive cells were performed as described in Material & Methods and expressed as number of cells per mm^2. The data are presented as scatter data plots (each point represents one individual) with mean ± SEM and analyzed with One-way ANOVA followed by Tukey's multiple comparison test.* P< 0.05, ** P<0.01*** P<0.001; NS: not significant

Supplementary Figure 6

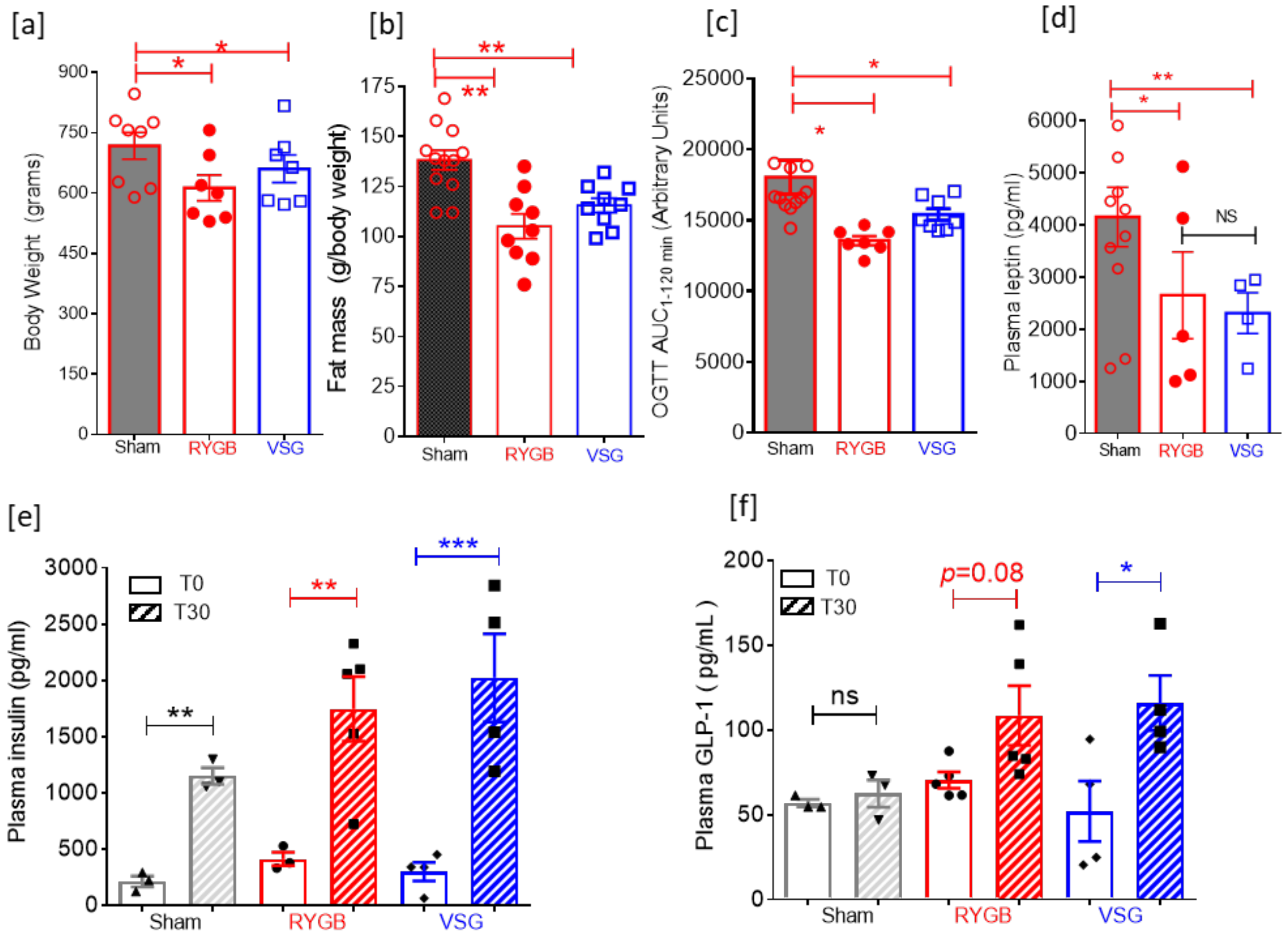

Characteristics of HFD fed rats before and after Sham-, RYGB- and VSG -surgeries.

[a] Body Weight **[b]** fat mass **[c]** calculated area under curve (OGTT AUC) of the time-dependent blood glucose levels after oral load of glucose. **[d]** Fasted plasma leptin levels. The data are presented as scatter data plots (each point represents one individual) with mean ± SEM and One-way ANOVA followed by Tukey's multiple comparison was used for statistical analyses. Plasma **[e]** insulin and **[f]** GLP-1 levels, 0 and 30 min after an oral load of glucose, 2 weeks after Sham-, RYGB- and VSG-surgery of HFD obese rats. Results are expressed as mean ± SEM and the statistical analysis was performed with Kruskall-Wallis Test. *P< 0.05, **P<0.01; *** P<0.001; ns for not significant.

Supplementary Text 1

HFD obese rats operated on RYGB or VSG exhibited significant reduction of their body weight, fat mass and have improved their oral glucose tolerance (Supplementary Fig. 6a-c). Plasma leptin levels (Supplementary Fig. 6d) were significantly reduced in RYGB- and VSG- HFD rats compared to sham-rats.

Consistent with the improvement of oral glucose tolerance, plasma insulin levels (Supplementary Fig. 6e) were further increased in RYGB– and VSG-operated HFD rats compared to sham-operated HFD rats. Moreover, plasma GLP-1 levels response to glucose that was blunted in sham-operated HFD rats, were significantly increased HFD rats operated on RYGB or VSG (Supplementary Fig. 6f). Collectively, the results recapitulate the beneficial effects of bariatric surgeries in obese subjects.

Supplementary Figure 7

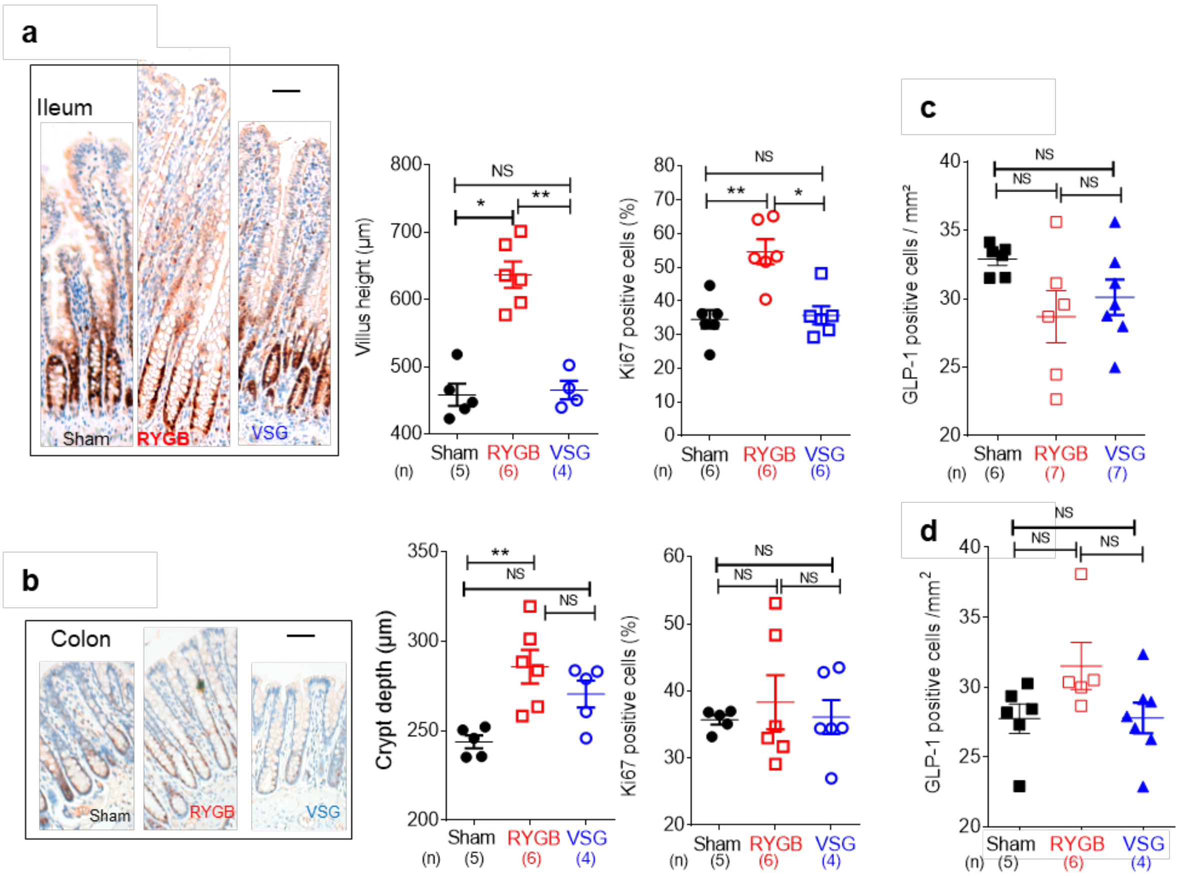

Morphological changes, Ki67 proliferative cells and GLP-1 positive cells [C & D] in the distal intestine of rats operated on Sham, RYGB or VSG. [a] Morphometric analyses showed an increase in villi height and in proliferating Ki-67 positive cells in the ileum after RYGB but not VSG. **[b]** In the colon, an increase in the crypt depth and a trend toward an increase in proliferating Ki67 cells was observed after RYGB but not VSG. Bar scale 100µm. **[c&d]** Quantification of the number of GLP-1 immunoreactive cells per mm^2 in the ileum **[c]** and the colon **[d]** of HFD obese rats operated on RYGB, VSG or sham- 2 weeks after surgery. Data are presented as scatter data plots (each point represents one animal) with mean ± SEM and were statistically analyzed with One-way followed by Tukey's multiple comparison test. *P <0.05, **P <0.01 *vs* sham. NS: not significant.

Supplementary Table 1

IDENTIFIED PEPTIDES FROM GCG

ILEUM									
Peptides sequences	Exogenous Protease	Q-value	Mascot score	MH+ (theoretical)	Position in Gcg	Endogenous protease signature	Domain	specific form signature	Abundance
[D].FINWLIQTKITD.[K]	Glu-C	0	51	1491,82059	[167-178]		Glp2	Gcg	1,80E+07
[D].NLATRDFINWLIQTKITD.[K]	Glu-C	0	17	2162,16043	[161-178]		Glp2	Gcg	1,70E+06
[E].DKRHSQGTFTSDYSKYLD.[S]	Glu-C	0	28	2147,99924	[50-67]		Glucagon	Gcg	2,60E+06
[F].TSDYSKYLD.[S]	Glu-C	7,97E-04	43	1091,48915	[59-67]	yes	Glucagon	Gcg	9,70E+06
[E].NARSFPASQTEPL.[E]	Glu-C	9,73E-04	30	1417,70702	[29-41]	yes	Glicentin-related polypepti	Gcg	1,10E+06
[Q].GTFTSDYSKYLD.[S]	Glu-C	9,73E-04	27	1396,6267	[56-67]	yes	Glucagon	Gcg	1,60E+06
[E].NARSFPASQTEPLED.[P]	Glu-C	1,24E-03	26	1661,77655	[29-43]		Glicentin-related polypepti	Gcg	2,20E+06
[D].FVQWLMNTKRNR.[N]	Glu-C	1,42E-03	26	1592,84783	[74-85]	yes	Glucagon	Gcg	3,50E+06
[D].FVQWLMNTKR.[N]	Glu-C	2,86E-03	19	1322,70379	[74-83]	PC1/3 (C-Term) [83-84]	Pro-Glp1	Pro-Glp1	3,40E+06
[E].FIAWLVKGRG.[R]	Glu-C	6,23E-03	14	1146,67822	[119-128]	protease activating Glp1 [128]	Glp1	Pro-Glp1 & Pro-Glp2	6,70E+05
[T].FTSDVSSYLEGQAAK.[E]	Lys-C	0	78	1602,76459	[103-117]	yes	Glp1	Gcg	2,20E+06
[F].TSDVSSYLEGQAAK.[E]	Lys-C	0	86	1455,69618	[104-117]	yes	Glp1	Gcg	1,30E+07
[M].NTILDNLATRDFINWLIQTK.[I]	Lys-C	0	44	2389,28742	[156-175]	yes	Glp2	Gcg	6,70E+06
[A].TRDFINWLIQTK.[I]	Lys-C	0	31	1534,83764	[164-175]	yes	Glp2	Gcg	1,00E+06
[R].DFINWLIQTK.[I]	Lys-C	0	60	1277,68885	[166-175]	yes	Glp2	Gcg	4,40E+06
[D].VSSYLEGQAAK.[E]	Lys-C	0	35	1152,58953	[107-117]	yes	Glp1	Gcg	6,90E+06
[E].GTFTSDVSSYLEGQAAK.[E]	Lys-C	0	19	1760,83373	[101-117]	yes	Glp1	Gcg	1,30E+07
[K].EFIAWLVK.[G]	Lys-C	0,0009674	33	1005,57678	[118-125]		Glp1	Gcg	6,20E+07
[K].YLDSRRAQDFVQWLMNTK.[R]	Lys-C	0	29	2271,1339	[65-82]		Glucagon	Gcg	1,90E+07
[K].RHSQGTFTSDYSKY.[L]	Lys-C	0	28	1676,76632	[52-65]	yes	Glucagon	Gcg	1,70E+05
[T].SDVSSYLEGQAAK.[E]	Lys-C	0	47	1354,6485	[105-117]	yes	Glp1	Gcg	1,30E+06
[A].EGTFTSDVSSYLEGQAAK.[E]	Lys-C	0	9	1889,87633	[100-117]	yes	Glp1	Gcg	5,30E+06
[L].ATRDFINWLIQTK.[I]	Lys-C	0,0003781	17	1605,87475	[163-175]	yes	Glp2	Gcg	2,40E+06
[R].RAQDFVQWLMNTK.[R]	Lys-C	0,007394	28	1636,82642	[70-82]	yes	Glucagon	Gcg	
[R].DFPEEVAIAEEL.[G]	Chymotrypsin	0	33	1361,6471	[131-142]	PC1/3 (N-term) [130-131]	Pro-Glp2	Pro-Glp1 & Pro-Glp2	3,20E+06
[Y].LEGQAAKEF.[I]	Chymotrypsin	0	58	992,50474	[111-119]		Glp1	Gcg	8,70E+06
[W].LIQTKITD.[K]	Chymotrypsin	0,0008565	34	931,54587	[171-178]	protease activating Gpl2 [178]	Glp2	Pro-Glp2	2,30E+07
[D].FPEEVAIAEEL.[G]	Chymotrypsin	0,004179	24	1246,62016	[132-142]	yes	Pro-Glp2	Gcg	4,60E+06
[R].HADGSFSDEMNTILDNLATR.[D]	Trypsin	0	27	2207,00334	[146-165]		Glp2	Gcg	9,70E+06
[R].DFPEEVAIAEELGR.[R]	Trypsin	0	51	1574,76968	[131-144]		Pro-Glp2	Gcg	2,00E+07
[K].EFIAWLVK.[G]	Trypsin	0	42	1005,57678	[118-125]		Glp1	Gcg	4,60E+07
[R].AQDFVQWLMNTK.[R]	Trypsin	0	50	1480,72531	[71-82]		Glucagon	Gcg	4,80E+07
[R].DFINWLIQTK.[I]	Trypsin	0	40	1277,68885	[166-175]		Glp2	Gcg	6,40E+07
[R].RAQDFVQWLMNTK.[R]	Trypsin	0,006556	28	1636,82642	[70-82]		Glucagon	Gcg	1,20E+07

GASTRIC MUCOSA									
Peptides sequences	Exogenous Protease	Q-value	Mascot score	MH+ (theoretical)	Position in Gcg	Endogenous Protease signature	Domain	specific form signature	Abundance
[R].DFINWLIQTK.[I]	Trypsin	4,80E-04	30	1277,68885	[166-175]		Glp2	Gcg	6,50E+05
[K].EFIAWLVK.[G]	Trypsin	8,07E-04	17	1005,57678	[118-125]		Glp1	Gcg	4,30E+05
[R].DFPEEVAIAEELGR.[R]	Trypsin	5,87E-04	8	1574,76968	[131-144]		Glp2	Gcg	3,40E+05
[D].FINWLIQTKITD.[K]	Glu-C	4,54E-02	10	1277,68885	[167-178]		Glp2	Gcg	6,30E+05

THEORETICAL PROTEASE CLEAVAGE SPECIFICITY

Proteases	cleavage after
Glu-C	D or E
Lys-C	K
chymotrypsin	F, L, W or Y
trypsin	K or R
endogenous PC1/3	KR or RR

GCG MEASURED ABUNDANCE

Dataset	ileum	gastric mucosa	Ratio ielum/gastric mucosa
Trypsin	5,30E+07	3,40E+05	155,8823529
GluC	1,00E+07	6,30E+05	15,87301587
LysC	2,80E+07		
Chymotrypsin	1,20E+07		

Identified peptides from GCG by LC-MS/MS in rat ileum and gastric mucosa. Peptides were generated by using different exogenous proteases (trypsin, Lys-C, Glu-C and chymotrypsin) with different cleavage specificities allowing to identify specific signatures of the presence of processed GCG. The Mascot database search engine was used to identify peptides with a "semi-cleavage" specificity parameter in order to impose exogenous protease cleavage rules at only one extremity of each peptide (the other extremity may be cleaved at any residue) and to identify specific signatures of endogenous proteases (like PC1/3). Peptide identifications were justified by Mascot scores (-10logP with P, the calculated probability that the observed match between the experimental data and the database sequence is a random event), Q-values (False discovery Rate) under 0.01 and theoretical mass MH+ (experimental mass) of each peptide. In addition, protein abundance measurements obtained by relative quantitate proteomics were used to give an estimation of GCG fold change between ileum and gastric mucosa conditions.

Supplementary Table 2: primer sequences

**Target gene**	**NCBI Reference**	**Forward primer**	**Reverse primer**
Rat *Gcg*	NM_012707.2	TGAGATGAACACGATTCTCGAT	AAGATGGTTGTGAATGGTGAAA
Rat *Pcsk1/3*	NM_017091.2	GGAGAGGAGTGGAAAAGATGG	GATTGCCATTCAGGCTGTTT
Rat *Pax6*	NP_037133.1	GCCCTCACCAACACGTACA	AGGTCTGACTGGGGACTGG
Rat *Pyy*	NM_001034080.1	TCCATCTCCTCCTGCTCATC	GAGCAGGACAAGCAGCATT

Supplementary Table 3 Characteristics of Patients for GLP-1 immunohistochemistry studies

	Patient N°.	BMI, kg/m^2	Indication for reoperation	Time after surgery, months
Obese control	1	41	N/A	N/A
	2	54.2	N/A	N/A
	3	49.8	N/A	N/A
	4	61	N/A	N/A
	5	56	N/A	N/A
	6	48	N/A	N/A
	7	51.6	N/A	N/A
	8	45.6	N/A	N/A
	9	40	N/A	NA
VSG group	1	39.7	Fistula	36
	2	42.3	dilatation	35
	3	40.4	fistula	48
	4	41.7	fistula	72
	5	33.7	dilatation	60
	6	32.3	fistula	53
RYGB Group	1	37.1	Persistent ulcer	18
	2	36.3	Persistent ulcer	25
	3	27.8	Pouch dilatation	48
	4	39.9	Persistent ulcer	53
	5	30.6	Pouch dilatation	45
	6	42.3	Persistent ulcer	19

Informed consent were obtained from all the 21 patients (15 females / 6 males). None of these patients has experienced intestinal obstruction or any other known issues that could have directly impacted the histologic characteristics of the samples. The embedded-paraffin tissue blocks of these patients treated by surgery in our Labeled Obesity Center at Bichat Hospital, Paris, (France), were retrospectively selected from the files of the Department of Pathology. Fundic mucosa sections were cut and used for histological and immunohistochemistry studies. N/A, not applicable
