# Supplementary material for: Bariatric surgery induces a new gastric mucosa phenotype with increased functional glucagon-like peptide-1 expressing cells
Source: Nat Commun. 2021 Jan 4;12:110. doi: 10.1038/s41467-020-20301-1 (PMC7782689; doi:10.1038/s41467-020-20301-1)
Supplement: Supplementary file 3 — Reporting Summary [file 41467_2020_20301_MOESM3_ESM.pdf]

Corresponding author(s): Andre BADO

Last updated by author(s): Oct 19, 2020

Reporting Summary

Nature Research wishes to improve the reproducibility of the work that we publish. This form provides structure for consistency and transparency in reporting. For further information on Nature Research policies, see our Editorial Policies and the Editorial Policy Checklist.

Statistics

For all statistical analyses, confirm that the following items are present in the figure legend, table legend, main text, or Methods section.

n/a	Confirmed	
☐	☒	The exact sample size (n) for each experimental group/condition, given as a discrete number and unit of measurement
☐	☒	A statement on whether measurements were taken from distinct samples or whether the same sample was measured repeatedly
☐	☒	The statistical test(s) used AND whether they are one- or two-sided<br>*Only common tests should be described solely by name; describe more complex techniques in the Methods section.*
☒	☐	A description of all covariates tested
☐	☒	A description of any assumptions or corrections, such as tests of normality and adjustment for multiple comparisons
☐	☒	A full description of the statistical parameters including central tendency (e.g. means) or other basic estimates (e.g. regression coefficient) AND variation (e.g. standard deviation) or associated estimates of uncertainty (e.g. confidence intervals)
☐	☒	For null hypothesis testing, the test statistic (e.g. F, t, r) with confidence intervals, effect sizes, degrees of freedom and P value noted<br>*Give P values as exact values whenever suitable.*
☒	☐	For Bayesian analysis, information on the choice of priors and Markov chain Monte Carlo settings
☒	☐	For hierarchical and complex designs, identification of the appropriate level for tests and full reporting of outcomes
☒	☐	Estimates of effect sizes (e.g. Cohen's d, Pearson's r), indicating how they were calculated

Our web collection on statistics for biologists contains articles on many of the points above.

Software and code

Policy information about availability of computer code

Data collection	cells counting Software Calopix version 4.1.0.1 powdered by TRIBVN
Data analysis	Data analysis using GaphPad version 6.00 for Windows , GraphPAd Software LA Jolla CAlifornia USA, www.graphpad.com

For manuscripts utilizing custom algorithms or software that are central to the research but not yet described in published literature, software must be made available to editors and reviewers. We strongly encourage code deposition in a community repository (e.g. GitHub). See the Nature Research guidelines for submitting code & software for further information.

Data

Policy information about availability of data

All manuscripts must include a data availability statement. This statement should provide the following information, where applicable:

- Accession codes, unique identifiers, or web links for publicly available datasets
- A list of figures that have associated raw data
- A description of any restrictions on data availability

The complete data of Mass Spectrometry analysis are available in the pride partner repository un the number PXD009867 (username: reviewer76595@ebi.ac.uk, pasword: WUvLvBYV) as .raw files, pepe.wml and XLSX files

Field-specific reporting

Please select the one below that is the best fit for your research. If you are not sure, read the appropriate sections before making your selection.

☒ Life sciences ☐ Behavioural & social sciences ☐ Ecological, evolutionary & environmental sciences

For a reference copy of the document with all sections, see nature.com/documents/nr-reporting-summary-flat.pdf

Life sciences study design

All studies must disclose on these points even when the disclosure is negative.

Sample size	The original plan was to get at least 4 animals per group for the in vivo experiment or 4 biologically independent samples for the in vitro experiments. Based on our previous experience, 4 samples were enough to detect a difference between 2 groups in the parmeters we generally measure.
Data exclusions	No data was excluded in this study
Replication	All experiments were performed using at least 2 replicates for in vitro. For in vivo all experiments have been repeated independently .
Randomization	HFD obese rats were randomly assigned to sham surgery or to sleeve gastrectomy or Roux-en-Y gastric bypass
Blinding	For the animal studies, the three anatomopathologists AS AC MH who have evaluated and quantified the immunostainings were blinded to group allocation.<br>For the human immunohsitochemistry, AC retrospectively selected the patients and paraffin blocks and only AS et MH examined the immunostainings slides and were blinded to the patients .

Reporting for specific materials, systems and methods

We require information from authors about some types of materials, experimental systems and methods used in many studies. Here, indicate whether each material, system or method listed is relevant to your study. If you are not sure if a list item applies to your research, read the appropriate section before selecting a response.

Materials & experimental systems

n/a	Involved in the study	
☐	☒	Antibodies
☒	☐	Eukaryotic cell lines
☒	☐	Palaeontology and archaeology
☐	☒	Animals and other organisms
☐	☒	Human research participants
☒	☐	Clinical data
☒	☐	Dual use research of concern

Methods

n/a	Involved in the study	
☒	☐	ChIP-seq
☒	☐	Flow cytometry
☒	☐	MRI-based neuroimaging

Antibodies

Antibodies used	Primary Antibodies Used :<br>Monoclonal mouse anti-Human Ki67 (# M7240; Agilent Dako); Clone MIB-1 dilutrion 1/100<br>Mouse monoclonal [colone number 8G9] to GLP-1 (amidated) antibody (#ab26278) (Abcam, Cambridge, UK MA) dilution 1/3000<br>5-HT (serotonin) Rabbit plolyclonal antibody (catalog #20080, lot #924005)(Immunostar WI, USA), dikution 1/10 000<br>Monoclonal mouse /human ghrelin antibody , monoclonal Rat IgG2A clone#883622 s #MAB8200, ((R&D systems) dilution 1/500<br>Human /Mouse Somatostatin antibody, Monoclonal Rat IgG1, clone# 906552 catalog# MAB2358(R&D systems) dilution 1/500<br>Secondary antibodies used are from Jackson ImmunoResearch Laboratory In; (Europe Ltd) and were used at dilution 1/300<br>Alexa Fluor® 488 AffiniPure Donkey Anti-Rat IgG, Cyanin Cy™3 AffiniPure Donkey Anti-Mouse IgG from
Validation	All the primary antibodies have been validated by the supliers (cf the supliers' website with the catalog number) and we tested different dilutions in our laboratory in order to choose the best working dilution. In addition, substitution of the primary antibody with PBS was used as a negative control.

Animals and other organisms

Policy information about studies involving animals; ARRIVE guidelines recommended for reporting animal research

Laboratory animals	8 weeks old Male Wistar rats from Elevage Janvier, France were used in this study

Wild animals	No wild animals were used in this study
Field-collected samples	No field-collected samples were used in the study
Ethics oversight	All animal studies followed ARRIVE guidelines and were conducted in compliance with EU directives for animal experimentation Institutional Animal care and were approved by the Ethical Committee of Paris North and The French Minister of Higher Education, Research and Innovation (MESRI Apafis #8290).

Note that full information on the approval of the study protocol must also be provided in the manuscript.

Human research participants

Policy information about studies involving human research participants

Population characteristics	For mucosal GLP-1 content :<br>Sixteen patients [12 females /4 males] of age median 42yr old [31-52; 95% Confidence Interval (CI), N=16], with median BMI of 43.85 [37.3-58.79; 95%CI, N=16].<br>For histology and immunohistochemistry studies, twenty one (21) patients treated by surgery were retrospectively selected from the files of the Department of Pathology, Bichat Hospital, Paris, France.<br>Obese-control median age 42yrs [35-51, 95%CI], VSG group median age 43.6 [35-52, 95%CI], RYGB group median age45yr [30-56, 95% CI]<br>Obese control group: median BMI 49.8 [41-56; 95% CI, N=9] , VSG group median BMI 40.05 ([32.3-42.3, 95%CI, N=6] RYGB group median BMI 36.70 ([27.8-42.3, 95%CI, N=6]
Recruitment	Obese subjects candidates for bariatric surgeries were recruited in the Integrated Center for Medical and Surgical Care of Obesity of Bichat hospital. They provided informed consent and during endoscopic examination, fundus and antrum biopsie were collected<br>For histology and immunohistochemistry studies, the parafin-embedded blocked from twenty one (21) patients that all gave informed consent were retrospectively selected from the files of the Department of Pathology, Bichat Hospital, Paris, France.
Ethics oversight	All patients gave a written informed consent and the study was approved by the Committee for the Protection of humans, Aulnay-sous-Bois, Seine Saint-Denis, France

Note that full information on the approval of the study protocol must also be provided in the manuscript.
